# microRNAs in Parkinson’s Disease: From Pathogenesis to Novel Diagnostic and Therapeutic Approaches

**DOI:** 10.3390/ijms18122698

**Published:** 2017-12-13

**Authors:** Loredana Leggio, Silvia Vivarelli, Francesca L’Episcopo, Cataldo Tirolo, Salvo Caniglia, Nunzio Testa, Bianca Marchetti, Nunzio Iraci

**Affiliations:** 1 Department of Biomedical and Biotechnological Sciences (BIOMETEC), University of Catania, Torre Biologica, Via S. Sofia 97, 95125 Catania, Italy; loredanaleggio@unict.it (L.L.); silvia.vivarelli7@gmail.com (S.V.); 2 Neuropharmacology Section, OASI Institute for Research and Care on Mental Retardation and Brain Aging (IRCCS), 94018 Troina, Italy; flepiscopo@oasi.en.it (F.L.); ctirolo@oasi.en.it (C.T.); scaniglia@oasi.en.it (S.C.); ntesta@oasi.en.it (N.T.)

**Keywords:** parkinson’s disease, microRNAs, biomarkers, exosomes, neuroprotective therapies

## Abstract

Parkinson’s disease (PD) is the most prevalent central nervous system (CNS) movement disorder and the second most common neurodegenerative disease overall. PD is characterized by the progressive loss of dopaminergic (DAergic) neurons in the substantia nigra pars compacta (SNpc) within the midbrain, accumulation of alpha-synuclein (α-SYN) in Lewy bodies and neurites and excessive neuroinflammation. The neurodegenerative processes typically begin decades before the appearance of clinical symptoms. Therefore, the diagnosis is achievable only when the majority of the relevant DAergic neurons have already died and for that reason available treatments are only palliative at best. The causes and mechanism(s) of this devastating disease are ill-defined but complex interactions between genetic susceptibility and environmental factors are considered major contributors to the etiology of PD. In addition to the role of classical gene mutations in PD, the importance of regulatory elements modulating gene expression has been increasingly recognized. One example is the critical role played by microRNAs (miRNAs) in the development and homeostasis of distinct populations of neurons within the CNS and, in particular, in the context of PD. Recent reports demonstrate how distinct miRNAs are involved in the regulation of PD genes, whereas profiling approaches are unveiling variations in the abundance of certain miRNAs possibly relevant either to the onset or to the progression of the disease. In this review, we provide an overview of the miRNAs recently found to be implicated in PD etiology, with particular focus on their potential relevance as PD biomarkers, as well as their possible use in PD targeted therapy.

## 1. Introduction

Parkinson’s disease (PD) is the second most common neurodegenerative disease after Alzheimer’s disease (AD), affecting approximately 1% of people over 65 years and 5% of those over 85 [1]. It has been estimated that a number of ~9 million of the population worldwide will develop PD by 2030 [2]. The main clinical hallmarks of PD affect motor functions, including resting tremor, rigidity and loss of postural reflexes [3]. Additionally, a number of non-motor signs, such as depression, sleep disorders [4], dementia and peripheral impairments [5] are recognized to precede and/or occur with the progressive loss of the dopaminergic (DAergic) neurons within the substantia nigra pars compacta (SNpc). This loss is coupled with the accumulation of protein aggregates of α-SYN into intraneuronal structures (called Lewy bodies and Lewy neurites) and a dysregulated immune activation in the SNpc, disrupting both neuron metabolism and neurotransmission [6,7,8]. As the disease progresses, the gradual loss of dopamine (DA) storage in the striatum results in decreased motor functions, coupled with the progressive impairment of autonomic, cognitive and behavioral functions [9,10,11]. One feature of PD is that the first motor disturbances are not observed until the loss of DAergic neurons in the SNpc reaches almost 70% and at least 80% loss of DA in the striatum. Accordingly, the preclinical phase of the progressive DAergic degeneration before the onset of symptoms is estimated to last 8–17 years, implicating the existence of compensatory mechanisms in early PD [12]. 

Therefore, the search for preclinical PD biomarkers represents a crucial goal to achieve in order to design future neuroprotective therapies for at-risk populations, aimed at delaying and/or limiting the ongoing degeneration process before the appearance of the first clinical symptoms. Additionally, this exploration might lead to the identification of novel molecular targets for the development of possibly more effective drugs for this devastating disease. Indeed, the causes and mechanisms contributing to DAergic degeneration are ill-defined [11] and, currently, there is no cure for PD but only treatments able to relieve the symptoms and to improve the quality of life. These treatments include: supportive therapies (such as rehabilitation, through the use of physiotherapy, occupational therapy and speech and language therapy) and palliative medications (e.g., the DA precursor levodopa (L-DOPA), dopamine agonists, catechol-O-methyl transferase (COMT) inhibitors, monoamine oxidase B (MAO-B) inhibitors, amantadine and anti-cholinergic molecules and immunomodulatory therapies) [13,14,15,16]. If the palliative drugs fail to adequately control patients’ symptoms, deep brain stimulation (DBS) can be used. DBS utilizes a surgically implanted neurostimulator able to deliver electrical stimulation to targeted areas in the brain that control movement, blocking the abnormal nerve signals that cause tremor, rigidity and walking problems [17]. 

While the above-mentioned approaches provide symptom relief for most patients and can be effective against PD motor symptoms for a number of years, adverse effects may arise over time, hence, compromising their actual efficacy [18]. The debilitating nature of PD and the lack of enduring therapies has motivated researchers to investigate cell-based approaches, via direct injection of relevant cell types into PD patients’ brains, to restore the DAergic neuronal loss. Such therapies include transplantation of fetal tissue (FT-T), embryonic stem cells (ES-T), or induced pluripotent stem cells (iPSC-T). While the FT-T is not a realistic route to clinical treatment in the future—for several ethical and logistical problems—ES-T and iPSC-T based approaches have an intrinsic potential in the cure of PD [19,20,21].

Familial PD cases, accounting for less than the 10% of PD, originate from mutations in α-SYN (*SNCA*), PARKIN (*PRKN*), ubiquitin C-terminal hydrolase L1 (*UCHL-1*), PTEN-induced putative kinase 1 (*PINK1*), protein deglycase (DJ-1) (*PARK7*) and leucine-rich repeat kinase 2 (LRRK2) (*PARK8*) genes. Oppositely, the majority of PD cases are so-called sporadic (idiopathic PD), thus underlying a critical interplay between genetic susceptibility and environmental factors [22,23,24]. In particular, aging, inflammation and exposure to neurotoxic agents have all been identified as pivotal contributors to the DAergic neuronal loss [24,25,26,27,28,29,30]. Both familial and idiopathic forms of PD share common molecular pathways, such as oxidative stress, mitochondrial impairment, neuroinflammation and disruption of the ubiquitin-proteasome machinery [11,31]. 

In several neurodegenerative diseases—including PD but also AD, Huntington’s disease (HD) and Tourette’s syndrome (TS)—dysregulation of non-coding RNAs (ncRNAs) levels have been reported [32,33,34,35,36,37,38]. ncRNAs are divided into small and long classes: small ncRNAs being shorter than 200 nt (e.g., miRNAs, piRNAs, snoRNAs, etc.) and long ncRNAs (lncRNA) between 200 nt to over 100 kb [39,40,41]. In 2007 Lukiw showed for the first time that a panel of miRNAs was altered in hippocampus from AD-affected patients [42]. Recently, other classes of ncRNAs were found also modified, such as the piRNAs, again in the context of AD [43] and the lncRNAs, in PD patients compared to controls [44]. Considering that miRNAs are the most studied class of ncRNAs, which play key roles in normal cellular physiology, as well as in pathogenesis of many diseases, in this review we will focus on the role of miRNAs in PD [45,46,47,48,49].

**Figure 1:**
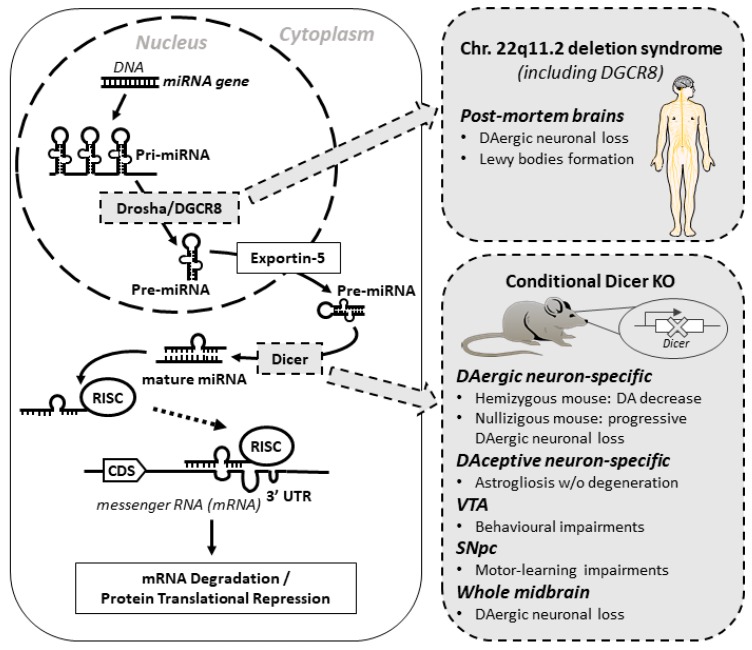
Biogenesis pathways of microRNAs and their dysregulations in Parkinson’s disease (PD). On the **left** panel, schematic representation of the intracellular miRNA canonical biogenesis, starting with transcription of pri-miRNA. The pri-miRNA is processed in the nucleus to pre-miRNA by the microprocessor complex (Drosha and DGCR8). The pre-miRNA is then exported via Exportin-5 into the cytoplasm where it is further cleaved by Dicer. Finally, the mature guide strand is assembled with members of the Argonaute family to form a functional RNA-induced silencing complex (RISC). On the right panels, ablation of DGCR8 (human) and its effect on brain (**top right**) and temporal and spatial controlled ablation of Dicer (mouse) and its effect on different brain areas (**bottom right**). knockout (KO); ventral tegmental area (VTA); coding sequence (CDS); substantia nigra pars compacta (SNpc).

miRNAs are ~22 nt in length and regulate the expression of their target genes by messenger RNA (mRNA) degradation or translational inhibition [50,51]. They act as post-transcriptional regulators by sequence complementarity either to the coding sequences (CDSs) or the UTRs of target mRNAs [52]. Many miRNAs work in close association with transcription factors and other components of the translational machinery modulating gene expression [53,54,55,56,57]. A miRNA is first transcribed as pri-miRNA, a longer primary transcript, which is cleaved by a nuclear complex containing DCGR8 and the RNAse III Drosha, to form the 70–100 nt precursor, called pre-miRNA. The pre-miRNA is then transported from the nucleus to the cytoplasm where the RNAse III Dicer cleaves the pre-miRNA into a 21–22 nt long duplex. One of the strands of the duplex associates with an Argonaute (AGO) protein within the RNA-induced silencing complex (RISC) and, finally, the mature miRNA can bind the target mRNA, regulating its expression (Figure 1).

Here, we provide an overview on the miRNAs associated with PD pathophysiology and involved in the regulation of PD-related genes. In addition, we will discuss the potential relevance of miRNAs as PD biomarkers and their possible development as innovative drugs in PD targeted therapy. 

## 2. miRNA Biogenesis Pathway and PD

Many studies demonstrated how Dicer and, consequently, miRNA pathway, are pivotal for the normal development, as mice deficient for Dicer are not viable and cannot survive beyond the embryonal stage [58,59]. For that reason, over the years, several conditional and tissue-specific Dicer-knockout (KO) mice models have been developed and characterized [60,61,62,63,64,65] (Figure 1). 

The first study correlating miRNA dysregulation and PD, following Dicer ablation, is from Kim et al. (see also Section 4) [53]. The authors generated a mouse homozygous Dicer conditional KO with CRE recombinase under the regulation of DA transporter promoter, thus leading to the specific deletion of Dicer in DAergic neurons. As an effect, these mice showed a progressive loss of midbrain DAergic neurons (appearing in two weeks-old mice and complete in six weeks-old mice). This midbrain DAergic neuron-specific loss was coupled with a dramatic reduction in mice mobility, suggesting that Dicer is essential for DAergic neurons differentiation and maintenance [53]. 

One year later, Cuellar and colleagues [66] generated a conditional Dicer KO, specifically within DA receptive (DAceptive) striatal neurons, by using a CRE recombinase expressed under the control of DA receptor promoter. These mice showed behavioral defects and decreased life span. Interestingly, the loss of Dicer in DAceptive striatal neurons led to astrogliosis, without any sign of neurodegeneration, thus highlighting the specific role of miRNA pathway in different subset of cells involved in PD [66].

In 2014, Pang and colleagues [67] examined the role of Dicer in adult mouse DAergic neurons, conditionally ablating Dicer expression in individual DAergic midbrain areas, such as the ventral tegmental area (VTA) and SNpc. They injected a viral CRE-expressing vector into specific brain areas of the conditional Dicer KO mice. Dicer KO in the VTA resulted in behavioral changes (e.g., hyperactivity), whereas in the SNpc induced motor-learning impairment. Only knocking out Dicer in the whole midbrain resulted in a progressive DAergic neuronal loss, in line with Kim’s observations. Importantly, this study supports the specificity of miRNA functionalities in distinct CNS brain regions, as well as across neuronal types [67]. 

More recently, Chmielarz and collaborators [68] characterized another mouse Dicer conditional KO, selectively in DAergic neurons of adult mice. They observed a dose-dependent effect, with PD-like symptoms heavier as the levels of Dicer decreased. In fact, while the hemizygous mice showed a marked decrease of DA levels in the striatum without any neuronal loss, the nullizygous mice showed a progressive DAergic neurodegeneration coupled with motoneuronal deficits, thus demonstrating that level of Dicer expressed was important in determining the severity of the PD-related symptoms (see also Section 4) [68].

Notably, another enzyme involved in miRNA biosynthesis (i.e., DGCR8) was found potentially related to PD. In fact, patients with a specific chromosomal deletion including *DGCR8* gene (called chromosome 22q11.2 deletion syndrome) showed a higher occurrence of PD in the adults carrying the deletion. Additionally, post-mortem brains analysis of those patients showed DAergic neuronal loss localized in the midbrain coupled with Lewy bodies detection. Functional experiments are further needed to directly correlate the PD occurrence with the *DGCR8* chromosomal loss [69] (Figure 1).

## 3. Regulation of PD-Related Genes Mediated by miRNAs

**Figure 2:**
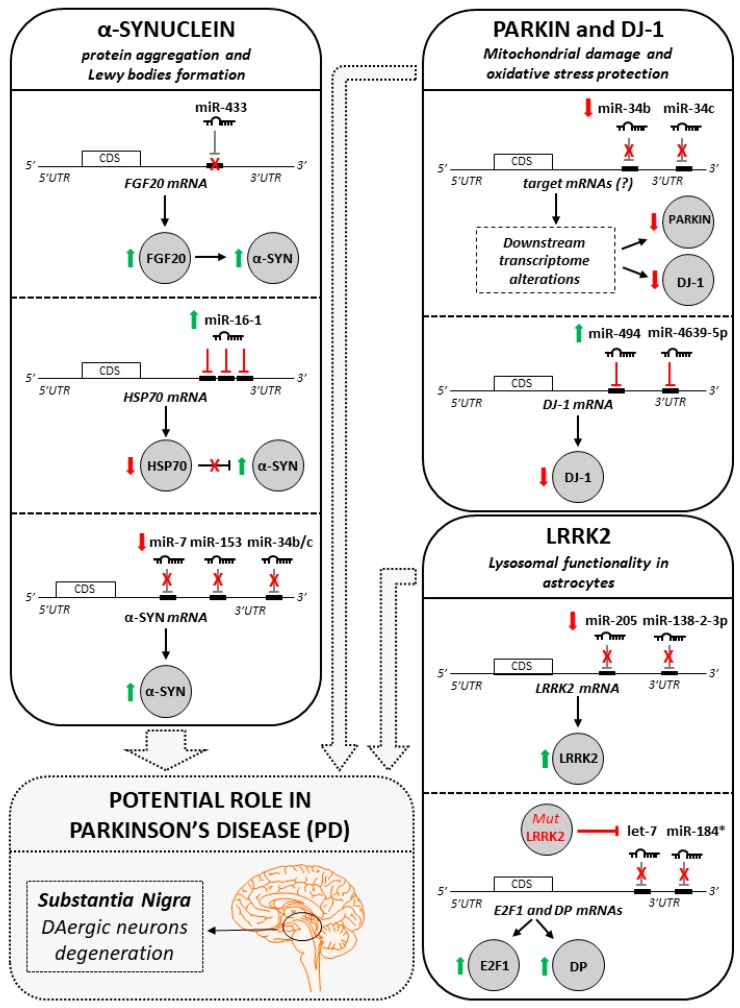
Regulation of PD-related genes mediated by miRNAs. Schematic representation of miRNA-mediated dysfunction networks in PD-related genes. Inhibitory arrows indicate how miRNAs act on their target sequence. Red crosses on inhibitory arrows indicate PD-related pathogenic processes blocking miRNA binding on their targets. Red crosses on miRNA target binding sites indicate an SNP interfering with the direct miRNA binding on their specific target sequence. Green and red thick arrows indicate, respectively, an upregulation or downregulation of a given miRNA or protein.

In addition to the studies aimed at characterizing the general role of miRNA machinery, many other studies investigated how specific miRNAs are able to target PD-related genes and, thus, to modulate their functions in different PD cellular and animal models. The miRNAs identified and their targets are shown in Figure 2.

### 3.1. miRNAs Targeting SNCA

α-SYN is a highly-conserved protein encoded by the *SNCA* gene and mainly expressed in neurons, where it is involved in clustering synaptic vesicles at the presynaptic terminals [70,71,72]. Furthermore, α-SYN contributes to the differentiation and survival of DAergic neuron progenitor cells [73,74]. α-SYN is an unstructured soluble protein, forming stably folded tetramers [75]. During pathological conditions, such as PD, α-SYN has been reported to aggregate forming insoluble fibrils, a hallmark of the Lewy bodies in DAergic damaged neurons [76,77]. 

To date, several miRNAs have been suggested as potential α-SYN regulators. Wang and colleagues in 2008 found a correlation between high levels of fibroblast growth factor 20 (FGF20) and increased susceptibility to PD, suggesting miR-433 as an important player, in vitro. The authors showed that FGF20 may indirectly trigger midbrain DAergic neuronal death, inducing chronically-elevated levels of α-SYN in human brain, thus conferring risk for PD. Importantly, through an in vitro luciferase reporter assay, the team assessed the ability of a specific single-nucleotide polymorphism (SNP) in the 3′UTR of FGF20 was able to impair miR-433 binding, suggesting that miR-433 could fail to repress FGF20 translation in PD patients carrying this SNP [78]. However, these data need further validation as subsequent reports have failed to prove a direct link between miR-433, FGF20 regulation, α-SYN and susceptibility to PD [79,80]. 

Following another route of investigation, Zhang et al. [81] found the link between the chaperone protein HSP70 dysregulation and α-SYN aggregation in a PD cellular model. They screened a set of miRNAs regulating HSP70 and, subsequently, found several miR-16-1 binding sites along the HSP70 3′UTR, thus suggesting miR-16-1 as the HSP70 main regulator. To corroborate the finding, the team used the miR-16-1 mimics to transfect the SH-SY5Y cells, observing high levels of α-SYN aggregation as a consequence of HSP70 downregulation [81].

Other miRNAs regulate α-SYN mRNA directly by binding its 3′UTR and negatively regulating its translation. Junn and collaborators published [82] a pioneering work where miR-7 has been suggested as a direct regulator of α-SYN expression. The 3′UTR of human *α-SYN* gene is twice longer than its CDS and contains several post-transcriptional regulatory elements. Using public prediction algorithms, the team found that miR-7 matches with the 119–127 nt region of *α-SYN* 3′UTR. They, therefore, transfected HEK293T cells with pre-miR-7 observing a reduction of α-SYN expression in a dose-dependent manner. They used a luciferase-*α-SYN*-3′UTR construct confirming miR-7 direct binding to *α-SYN* 3′UTR. Furthermore, in a MPTP PD mouse model, they observed miR-7 reduced levels in the SNpc, correlated to nigrostriatal system neurodegeneration and α-SYN upregulation [82]. 

Later on, Doxakis and colleagues [83] further clarified the roles of miR-7 and miR-153 in downregulating α-SYN, both at the transcript and protein level. They found that miR-7, miR-153 and α-SYN levels were higher specifically in cultured neurons, suggesting the two miRNAs as α-SYN expression modulators. Given the high levels of miR-7 and miR-153 expressed in the mouse midbrain, their deregulation may be important in PD onset [83].

Next, the relevance of miR-7 and miR-153 in PD was further confirmed by other reports. Choi and colleagues demonstrated that miR-7 was able to protect several cell types (SH-SY5Y cells, differentiated human progenitor ReNcells, ventral midbrain (VM) cells and primary mouse neurons) against the active metabolite of the environmental PD neurotoxin MPTP/MPP^+^, recognized to induce DAergic toxicity [84]. Subsequently, Fragkouli and colleagues reported that miR-153 overexpression reduced MPP^+^-induced neurotoxicity in murine DAergic neurons [85].

Two other miRNAs—directly acting on α-SYN—were identified by Kabaria and colleagues [86]. The group found in an in vitro SH-SY5Y PD cellular model that miR-34b and miR-34c were able to bind α-SYN 3′UTR reducing its expression. On the contrary, inhibition of miR-34b/c led to increasing expression of α-SYN and formation of α-SYN aggregates. Moreover, they found a polymorphism within α-SYN 3′UTR interfering with miR-34b binding, thus leading to α-SYN overexpression. However, the association of this polymorphism with PD risk needs still to be clarified with further studies [86]. 

### 3.2. miRNAs Targeting PRKN and PARK7

*PRKN* and *PARK7* genes, encoding respectively for the proteins PARKIN and DJ-1, are both associated with the pathogenesis of autosomal recessive PD [87]. PARKIN protein is expressed in both neuronal and non-neuronal cells. It has been reported that PARKIN participates in the proteasome-mediated degradation, together with the ubiquitin-conjugating enzyme UBCH7 [88,89]. *PRKN* mutations cause autosomal recessive juvenile parkinsonism (AR-JP). The AR-JP form of PD is correlated with the loss of ubiquitin-protein ligase activity, indicating that mutations in *PRKN* gene cause PD insurgence [90]. PARKIN has been also found expressed in mitochondria, where it binds mtDNA and protects against mtDNA damage in oxidative conditions, inducing repair mechanisms [91,92]. DJ-1 protein instead is encoded by the *PARK7* gene and represents another important protein involved in autosomal recessive primary PD. DJ-1 is a protein of the peptidase C56 family, thought to be an oxidative sensor and playing a causal role in cellular oxidative stress response. In fact, mutations in *PARK7* gene lead to PD due to the increased sensibility to ROS-mediated neuronal damage [93]. Moreover, several data indicate that DJ-1 binds PARKIN protein during oxidative stress, protecting mitochondria from oxidative stress [94].

miR-34b and miR-34c were found to be downregulated in PD patients and specifically in the amygdala, SNpc, frontal cortex and cerebellum, coupled with a significant decrease in the concentrations of PARKIN and DJ-1 proteins [95] (see Section 4). Performing in vitro studies, the depletion of miR-34b/c in differentiated SH-SY5Y neuroblastoma line resulted in cell death associated with impaired mitochondrial function and oxidative stress. Considering that expression of target genes is expected to increase upon the downregulation of their respective miRNAs, it is likely that miR-34b and miR-34c do not directly target PARKIN and DJ-1 mRNA [95].

DJ-1 protein has been found reduced also in the SNpc of sporadic PD patients. Xiong and colleagues [96] found that DJ-1 expression is post-transcriptionally regulated by miR-494. This miRNA was able to bind *DJ-1* 3′UTR, reducing its expression. The authors demonstrated that the overexpression of miR-494 exacerbates the MPTP-induced neurodegeneration, via downregulation of DJ-1 [96].

A recent work performed by Chen and colleagues in 2017 showed that DJ-1 is a direct target of miR-4639-5p. Interestingly, this miRNA was found significantly upregulated in plasma from PD patients (see also Section 5.1). Through in vitro studies conducted on HEK293T and SH-SY5Y cells, the authors confirmed the ability of miR-4639-5p to directly bind DJ-1 transcript at its 3′UTR and, as a result, DJ-1 protein was downregulated [97].

### 3.3. miRNAs Targeting PARK8

LRRK2 is an unusually large protein (2527 amino acids) that in humans is encoded by the *PARK8* gene. LRRK2 is a kinase belonging to the ROCO (Roc/COR) superfamily which is characterized by the presence of tandem Ras of complex (Roc) G-domain, kinase domains and carboxy-terminal of Roc (COR) sequence which links them [98]. Gain-of-function mutations in the *PARK8* gene can cause either familiar or sporadic PD [98,99,100,101]. The molecular mechanism of LRRK2 action is not completely uncovered. It has been shown that LRRK2 interacts in vitro with PARKIN and the expression of mutant LRRK2 induced apoptotic cell death in neuroblastoma cells and in mouse cortical neurons [102]. Very recently it has been also observed how LRRK2 is able to modulate DAergic receptors vesicle trafficking in SHSY-5Y cells as well as in primary striatal neurons [103].

In 2013, it has been found a miRNA able to directly regulate LRRK2 expression. Cho and collaborators [104] evaluated LRRK2 expression levels in the frontal cortex of PD and PDD (PD with dementia) patients compared with healthy controls. In both PD and PDD patients LRRK2 levels were higher than controls, although LRRK2 transcript levels were comparable between each other. To explain this discrepancy, the authors analyzed the 3′UTR of *LRRK2*, finding a miR-205 target site. Moreover, they showed a significant inverse correlation between LRRK2 and miR-205 levels, with high LRRK2 and low miR-205 in PD and PDD. Notably, upon overexpression of miR-205 in cell lines and primary neuron cultures, LRRK2 was found to be downregulated, thus possibly preventing its detrimental effects in the brains of PD patients [104].

One year later, Cardo and colleagues [105] correlated the variability of the LRRK2 3′UTR sequence with the risk of PD development. They screened a cohort of 743 PD patients and 523 healthy controls, finding a total of 12 variants within the LRRK2 3′UTR. In particular, by using nine post-mortem SNpc patient samples, the authors validated a SNP linked to PD and predicted to alter miR-138-2-3p binding. This miRNA was showed to be expressed in all the SNpc brain samples, although a larger number of brain tissues is required to verify its genetic association with LRRK2 expression [105].

In addition to the growing body of knowledge, showing that specific miRNAs directly or indirectly modulate the expression of disease-associated genes, LRRK2 has been characterized as a disease-linked protein able to regulate the miRNA pathway. In fact, in 2010 Gehrke and colleagues [106] demonstrated that pathogenic LRRK2 (i.e., I1915T or G2019S mutants) negatively regulated miRNA-mediated translational repression in *Drosophila melanogaster* brains, with toxic effects on DAergic neurons. In particular, they found that pathogenic LRRK2 was able to upregulate E2F transcription factor 1 (E2F1) and dimerization partner transcription factor (DP) levels, which in turn may lead to abortive cell division and neuronal cell death. Interestingly, the authors elucidated this mechanism, showing that G2019S LRRK2 inhibits the expression of let-7 and miR-184*, two miRNAs targeting E2F1 and DP, thus increasing their expression. On the other hand, the upregulation of let-7 and miR-184 * attenuated the neurotoxic effects of mutant LRRK2. These findings suggest that LRRK2 may play a role in PD pathogenesis via miRNA pathway modulation, therefore, highlighting new possible therapeutic strategies for PD [106]. 

### 3.4. miRNAs Targeting Genes Involved in Neuroinflammation

As previously introduced, neuroinflammation is a major hallmark of PD [11,25,28] and high levels of proinflammatory cytokines IL-1β, TNF-α, IL-6 and INF-γ, are produced in PD brains, as well as in MPTP-treated mouse models [107]. Recently, a growing number of miRNAs were studied in an attempt to identify potential regulators of glial inflammatory response in PD.

In 2015, Prajapati and collaborators [108] found that TNF-α was able to both trigger cell death in SH-SY5Y cells, as well as to sensitize SH-SY5Y to apoptosis in the presence of different PD stress conditions (i.e., MPTP, 6-OHDA, Rotenone). The authors measured the expression levels of miRNAs and their mRNA targets in TNF-α-treated SH-SY5Y cells and found nine miRNAs upregulated (let-7b, let-7g, miR-103, miR-155, miR-16-5p, miR-17, miR-204, miR-27 and miR-98) and seven miRNAs downregulated (let-7a, miR-128, miR-145, miR-181a, miR23a, miR-23b and miR-320a). Importantly, they found that putative targets of upregulated miRNAs were involved in three neuronal-specific pathways, such as neuronal differentiation, axonal guidance and nerve projection development. Moreover, they showed that TNF-α regulates miRNAs targeting mitochondrial complex-I and complex-V respiratory subunits, critical for neuronal functionality. In fact, ATP5G3—a subunit of F1-ATP synthase whose expression is decreased in the presence of TNF-α—is a target of both miR-155 and miR-27. To confirm the role of miR-155, they transfected SH-SY5Y cells with antago-miR-155, obtaining a decrease in the cell death in the presence of TNF-α. This study supports the role of TNF-α as regulator of miRNAs targeting mitochondrial functions, which in turn may cause DAergic neuronal loss [108]. 

In particular, miR-155 was previously shown to play a key role in the regulation of inflammatory processes. For example, miR-155 (induced by LPS, INF-γ and TNF-α) inhibited FADD, SOC1, IKK, IL13Rα1 and SMAD2. In turn, this inhibition resulted in upregulation of the proinflammatory molecules IL-1, IL-6, TNF-α and inducible nitric oxide synthase (iNOS) [109,110,111,112]. In 2016, Thome and colleagues analyzed the expression of 84 inflammation- and autoimmune-associated miRNAs in a PD mouse model overexpressing α-SYN (AAV2-SYN transduced mice). They observed miR-155 upregulation at both two and four weeks after virus transduction, compared to controls. To evaluate the involvement of miR-155 in the inflammation and neurodegeneration, they used miR-155 knockout mouse model (miR-155^−/−^ mouse). The lack of miR-155 prevented: (i) the increase of major histocompatibility complex II (MHCII), important marker of reactive microgliosis; and (ii) the loss of DA neurons, triggered by the α-SYN overexpression. Notably, rescuing miR-155 by using miR-155 mimics, reconstituted the inflammatory response to α-SYN fibrils. In conclusion, those results uncovered the central role of miR-155 in the microglial inflammatory response to α-SYN related neurodegeneration, suggesting miR-155 as a potential therapeutic target for regulating the inflammatory response in PD [113] (see also Section 6). 

Another miRNA—miR-7, previously reported to regulate α-SYN expression in DA neurons [82]—is recently emerging in the context of neuroinflammation. Zhou and colleagues [114] found that the inflammasome (nod-like receptor protein 3 gene (*NRLP3*)), expressed in microglial monocytes, is a direct miR-7 target. Importantly, injecting miR-7 mimics directly into mouse striatum, suppressed NLRP3 inflammasome activation and attenuated DAergic neuronal cell death in the MPTP mouse model of PD. This new link between miR-7 and NLRP3 inflammasome-mediated neuroinflammation in MPTP-induced nigrostriatal toxicity have lead the authors to suggest a potential therapeutic implication for the modulation of miR-7 in the context of NLP3 inflammasome activation-mediated DAergic neuron death [114] (see also Section 6). 

Furthermore, recent studies from He and colleagues [115] showed a role for miR-7116-5p in MPP^+^- induced microglial inflammatory response. Here, in an in vitro microglial cell culture model, MPP^+^ potentiated TNF-α production via a downregulation of miR-7116-5p. Accordingly, elevating the expression of miR-7116-5p in microglia prevented the overproduction of TNF-α and reduced loss of DA neurons, in vivo, in the mouse MPTP model [115]. The authors suggested a model whereby in addition to its direct inhibitory effects on neuronal survival, MPP^+^ could also damage the DA neurons by potentiating inflammation (i.e., TNF-α)-dependent DAergic neurodegeneration via miR-7116-5p inhibition [115]. 

In summary, amongst the studied miRNA, miR-155, miR-7 and miR-7116-5p were shown to either upregulate (miR-155) or downregulate (miR-7, miR-7116-5p) the inflammatory response in cellular in vitro and/or in vivo PD neurotoxic models, which are associated to either death or survival of DAergic neurons. Future studies are needed to better link specific miRNAs/mRNAs to the astroglial cell response to inflammation and neurodegeneration in PD models. This aspect appears of specific interest in light of the study of Nair and collaborators, showing that the differentially expressed miRNAs found in post-mortem PD-striatum were associated to the inflammatory response. In particular, using a pathway predictive analysis tool, the authors found that the majority of the predicted altered transcripts (as a consequence of miRNA dysregulation), were significantly associated with NF-κB pro-inflammatory network, in turn linked with neuronal signaling and stress response (see also Section 4) [116]. Altogether these studies may lead to the identification of new druggable targets for downregulating microglial activation and possibly mitigate DAergic neuron death in PD (see also Section 6).

### 3.5. PD-Related miRNAs in Other Alpha-Synucleinopathies and Neurodegenerative Diseases

Multiple system atrophy (MSA) is considered a Parkinsonian Syndrome, together with progressive supranuclear palsy (PSP), corticobasal degeneration (CBD), dementia with Lewy bodies (DLB) and PD [117]. MSA is a progressive neurodegenerative disease characterized by α-SYN aggregates in oligodendrocytes, called glial cytoplasmic inclusions (GCIs). 

In 2016 Schafferer and colleagues analyzed the miRNA-mRNA network in a mouse model of MSA [118], recapitulating the early pre-motor phase of the disease with the presence of GCIs. Through miRNA next generation sequencing (RNA-seq), they identified 59 differentially-expressed miRNAs in the SNpc and 33 in the striatum of MSA mice. Among those, miR-433 showed specific downregulation in the MSA striatum and was previously found downregulated also in the cerebellum of post-mortem MSA brains [119]. This miRNA regulates the expression of HDAC6, a histone deacetylase involved in autophagy regulation and co-localizing with α-SYN into the CGIs [120,121]. Interestingly, miR-433 was previously proposed as FGF20 regulator in PD, thus possibly playing a dual role in both MSA and PD (see Section 3.1). In addition, the authors also observed miR-19b as significantly downregulated in MSA mice [118]. Accordingly, Fernandez-Santiago and co-workers found miR-19b downregulated in the prodromal stage of alpha-synucleinopathies and pinpointed this miRNA as a potential biomarker also for PD and DLB (see also Section 5) [122].

Three miRNAs, miR-132, miR-124 and miR-34, have been linked to AD, PD and HD by several studies, as their target genes have been found potentially involved in cognitive functions and neuronal maintenance [123,124,125,126,127,128,129,130]. In particular, miR-132 was found downregulated in all three diseases. This miRNA targets both MeCP2 (methyl CpG-binding protein2) and SIRT1 (NAD-dependent protein deacetylase sirtuin-1), involved in the molecular mechanisms of learning and memory, as well as neuronal maturation and protection [123,131,132,133,134,135]. As a consequence, miR-132 KO mice displayed deficits in learning and memory [136]. Moreover, in the triple transgenic AD (3× Tg-AD) mouse model, miR-132/212 deletion enhanced memory deficits through a direct impairment of protein Tau, leading to pathological aggregation and consequent cholinergic neurodegeneration in vivo [137]. Importantly, miR-132 was found to negatively regulate DAergic neuron differentiation in mouse-derived ES cells, by directly suppressing Nurr1 (nuclear receptor related 1 protein) expression [138]. Nurr1 have been associated with disorders linked to DAergic dysfunction, including PD [139,140]. Notably, miR-132 was found dysregulated in different specimens from post-mortem PD samples (see also Section 4 and Section 5).

Additionally, mir-124 is highly expressed in the mammalian brain [125,141] and found downregulated in AD, PD and HD [37]. miR-124 is one of the most abundant miRNA expressed in the adult brain [142] and it is involved in preserving the neuronal identity and synaptic plasticity [143,144,145,146]. Interestingly, miR-124 is linked with PD. In fact, a significant decrease of miR-124 has been reported in both the SN of MPTP-treated mice as well as in DAergic neurons in vitro, while its overexpression improved cell survival [147,148]. Therefore, miR-124 has been proposed as potential PD therapeutic target (see Section 6). 

miR-34 family includes three members, miR-34a, miR-34b and miR-34c, sharing similar seed sequences and found to be dysregulated in AD, HD and PD (see also Section 3.1, Section 3.2 and Section 4). miR-34a/c were found upregulated in AD, miR-34b increased in HD and miR-34b/c were highly downregulated in PD. As mentioned above, miR-34b/c inhibition leads to a direct α-SYN accumulation [86], as well as to indirect downregulation of PARKIN and DJ-1 proteins [95]. Moreover, like miR-132, both miR-34a and miR-34c were reported to target SIRT1, a pivotal neuroprotective protein [149,150,151].

In summary, these studies support the concept that different neurodegenerative diseases share pathogenic pathways in which specific miRNAs may play a common role in regulating neurodegeneration. A better understanding of such pathways and how they are interconnected will possibly expand the number of miRNAs useful as novel biomarkers and new targets for treatment (see Section 5 and Section 6).

## 4. miRNAs in Post-Mortem PD Brain-Derived Samples

Over the last decade, many studies have been directed towards the identification of a specific miRNA signature in PD, with the final goal of uncovering which miRNAs may be functionally relevant for either the onset and/or the progression of the disease [152]. As discussed in Section 2, the first study on miRNAs in PD has been carried out by Kim and colleagues in 2007. In the same work, the authors also analyzed a panel of 230 miRNA precursors within the midbrain, cortex and cerebellum from PD patients compared to healthy controls. Amongst the miRNAs analyzed, miR-133b has been found specifically deficient in PD samples. The authors further validated their findings in rodent models, in vivo, using (i) adult Aphakia mice deficient for the transcription factor PITX3, which phenotypically shows a selective embryonic degeneration of dopamine neurons within the SN and, to a smaller extent, in the VTA; and (ii) mice treated with the dopamine neuron-specific toxin 6-hydroxydopamine (6-OHDA) [53]. Hence, while miR-133b was specifically expressed in midbrain of normal mice, as in humans, the expression was dramatically reduced in both rodent DA deficiency models. Additionally, they identified a negative feedback loop where *PITX3* is a miR-133b direct target, whereas PITX3 specifically induces the transcription of miR-133b [53].

Although this original study paved the way for a whole new field of research, several following studies did not further confirm miR-133b dysregulation as causal in PD neurodegeneration, neither in rodents nor in human post-mortem brain samples. In fact, in 2012 Heyer and colleagues [153] evaluated the role of miR-133b in vivo, generating and characterizing the miR-133b null mouse. They observed: (i) the PITX3 expression levels; (ii) the DAergic neurons number; and (iii) the general midbrain and striatum morphology were all preserved in miR-133b KO mice compared to wild type (WT), importantly, in both within young and elderly mice, thereby suggesting that miR-133b does not play a significant role in DAergic neuron development and maintenance in vivo [153]. One explanation could be the existence of paralogues miR-133a1 and miR-133a2 expressed, as well, in the midbrain that differs for only one base and may compensate for miR-133b loss in the KO mice [153]. MiR-133a1 and miR-133a2 double-KO mice have recently been reported [154], hence, it would be possible, in principle, to generate a triple miR-133 KO mouse to determine whether the whole miR-133 family is key for brain development and misfunctioning in PD [153]. In line with Heyer’s study, Schalaudraff and colleagues [155] analyzed the expression pattern of both mRNAs and miRNAs in five sporadic PD brains compared to eight healthy controls, by using an optimized single-DA-neuron-cell qPCR technique. Although they found elevated mRNA levels of DAergic release-related genes and PARK genes in DAergic neurons from sporadic PD patients, miR-133b levels were found unaltered, thus underlining the lack of causal connection between miR-133b dysregulation and PD etiology in vivo [155]. However, it is not possible to conclusively exclude that a decrease in miR-133b may enhance DA neurons’ susceptibility to neurotoxin-mediated PD neurodegeneration and this issue needs to be investigated further [153].

Many other groups tried to better clarify which miRNA(s) may be functionally involved in PD. In 2011, Miñones-Moyano and colleagues [95] evaluated miRNA expression in 11 PD patients in the motor stage of the disease vs. 6 controls, using a miRNA array and qPCR validation. They found a decreased expression of miR-34b/c in brain areas including the amygdala, frontal cortex, SN and cerebellum. Furthermore, deregulation of miR-34b/c was detected already in the pre-motor stages of PD, in never-treated patients. They further characterized the mechanism underneath, by using an in vitro model of PD, finding that miR-34b/c downregulation was associated with a decrease in the expression of DJ-1 and PARKIN, two proteins involved in both familiar and idiopathic PD. In line with the in vitro results, DJ-1 and PARKIN expression was reduced in those PD brain samples displaying strong miR-34b/c downregulation (see also Section 3.1, Section 3.2 and Section 3.5) [95].

Cardo and colleagues [156] evaluated the miRNAs expression pattern in the SN of 8 post-mortem PD and 4 healthy subjects. By using a TaqMan low-density array (TLDA) (TaqMan, Foster City, CA) with 733 miRNA-probes and subsequent qPCR validation, they found 11 miRNAs deregulated in PD samples vs. controls. Although some of those (i.e., miR-339-5p, miR-198, miR-485-5p and miR-548d) were previously found altered in other neurodegenerative diseases [157,158,159,160,161], they detected a highly heterogeneous profile between PD patients, with no miRNA as an unequivocal indicator [156].

The same year, Kim and collaborators [162] compared the post-mortem miRNA expression of 8 sporadic PD vs. 8 healthy controls, by using human microRNA TaqMan array on total RNA derived from laser-captured DAergic neurons. They observed a distinctive miRNA expression profile in DAergic neurons, that is dysregulated in PD. Interestingly they found an upregulation of miR-126, in association with dysregulated IGF-1/PI3K/AKT signaling in PD DAergic neurons (see also Section 6) [162].

The following year, Briggs and colleagues [163] compared the miRNA expression of 8 idiopathic PD vs. 8 controls, as well as males vs. females, post-mortem SNpc brain region. miRNA profiling was performed using again human microRNA TaqMan array. They showed that several miRNAs were dysregulated in PD neurons and differentially expressed between male and female samples, with more upregulated miRNAs in males and more downregulated miRNAs in females. In particular, 14 significantly dysregulated miRNAs were found to correlate with 16 PD-associated genes, linked with various aspects of PD pathogenesis. However, after a careful bioinformatics analysis, except for miR-132 and miR-184, the authors could not identify statistically significant dysregulated PD-specific miRNAs [163].

Another brain region affected in PD is the striatum, receiving glutamatergic and dopaminergic inputs from several areas of the brain and regulating voluntary movements. As anticipated in Section 3.4, Nair and colleagues in 2016 analyzed miRNA expression changes in post-mortem PD striatum, particularly in PD putamen tissues, using a miRNA Expression Array kit with 800 probes. They analyzed 12 PD samples compared to 12 healthy controls, finding 13 dysregulated miRNAs, six of which found significantly upregulated (miR-3195, miR-204-5p, miR-485-3p, miR-221-3p, miR-95 and miR-425-5p) and seven downregulated (miR-155-5p, miR-219-2-3p, miR-3200-3p, miR-423-5p, miR-4421, miR-421 and miR-382-5p). Computational analysis showed that many of these miRNAs were associated with inflammatory response and other mechanisms activated by oxidative stress in PD striatum. Considering that almost all PD patients in this study were treated with L-DOPA, the authors suggested that miRNAs found dysregulated in PD, might mediate antioxidant effect of the drug treatment, by suppressing proinflammatory factors and upregulating antioxidant factors [116].

Finally, as mentioned in the Section 2, Chmielarz and colleagues [68] in 2017 further characterized the role of Dicer in the regulation of the miRNA/mRNA network during aging mice. They applied TaqMan qPCR-based miRNAs arrays on DAergic laser microdissected neurons from aged vs. young mice. The authors found Dicer specifically downregulated in the ventral midbrain of aged WT mice and, as consequence, 42 downregulated mature miRNAs, whereas their precursors were unchanged. This finding corroborated the hypothesis that the age-dependent miRNAs deregulation was a consequence of the impaired miRNAs processing [68].

In summary, the studies herein presented have disclosed some of the miRNAs that may have a link with PD pathophysiology and thus require further analyses in more extended PD patient populations to more precisely identify potential disease candidates. However, it should be noticed that post-mortem studies represent the endpoint of the disease, after a long clinical history. Indeed, miRNA expression may vary according to the progression of PD, the specific stage of the disease and/or the different treatments used, thus explaining the heterogeneity of the miRNAs identified [164]. In order to assess miRNA profiles in PD patients, while the disease arises and progresses, researchers have searched for methodologies to detect miRNA in liquid specimens from PD patients, therefore allowing for the identification of potential PD biomarkers useful for diagnosis and prognosis of the pathology.

## 5. miRNAs as Biomarkers for PD Diagnosis

PD is a complex and heterogeneous condition resulting in movement deficits coupled with cognitive impairments as well as additional peripheral symptoms. Currently, the differential diagnosis is based on clinical signs and motor functions rating. The main issue is that the rating can be biased and it is performed only once the DAergic neuron loss is already around 70% [165]. 

Since there is no reliable quantitative diagnostic test for PD, molecular biomarkers can be potential clinical tools to ease early and accurate PD diagnosis. Current PD candidate biomarkers are based on PD-related proteins detection in cerebral spinal fluid (CSF) and brain tissue, such as α-SYN for protein aggregation and Lewy body formation or DJ-1 for mitochondrial dysfunction [166]. The result is that sample collection can be invasive (CSF) or possible only post-mortem (brain tissue). In contrast, blood is an ideal source for biomarkers being easy and quick to sample from patients. Plasma-based biomarkers were discovered for many diseases such as cancers, HD and heart-related diseases [167,168,169]. MiRNAs present in plasma are known to be abundant, tissue-specific, highly stable and quantifiable. Circulating miRNAs can be thus characterized and possibly used as non-invasive biomarkers, facilitating the early detection of PD as well as monitoring the progression of the pathology [170]. A list of miRNAs potentially useful for PD diagnosis is shown in Supplementary Table S1.

### 5.1. miRNAs in Peripheral Blood Samples

Margis and colleagues in 2011 ran the first study on peripheral blood (PB) from treated and untreated PD patients, showing that miR-1, miR-22* and miR-29a were downregulated, while miR-16-2*, miR-26a and miR-30a were increased in L-DOPA-treated PD patients, thus suggesting the possibility to distinguish between treated and untreated patients [171]. 

The same year, Martins and collaborators [172], carried a miRNA expression profiling study in peripheral blood mononuclear cells (PBMCs) derived from 19 patients and 13 controls. They found 18 miRNAs differentially expressed between the two groups. All of these 18 miRNAs were under-expressed in patients. Using Ingenuity Pathway Analysis (IPA) database, the authors performed association studies leading to the identification of two pathways relevant in PD (i.e., the glycosphingolipid biosynthesis and the protein ubiquitination). Finally, they identified miR-30b, miR-30c and miR-26a, as main modulators of these two pathways potentially associated with PD susceptibility [172]. 

Later on, Khoo and colleagues [173] acquired a global miRNA expression analysis from a panel of 32 PD patients and 32 controls using microarrays. As a result, following a validation step via qPCR, they finally identified four best candidates: miR-1826, miR-450b-3p, miR-626 and miR-505. The group performed a further validation in a different set of blood samples from 30 PD patients and 8 controls. However, low predictive values were shown in the second validation set, meaning that even if there was a theoretical feasibility of obtaining PD biomarker candidates from plasma circulating miRNAs, they could not validate biomarker candidates, due to clinical and sample variability [173]. 

In 2013, Soreq and colleagues [174] investigated on miRNA expression in leukocytes of 7 PD patients and 6 healthy controls, before and after DBS treatment, by using small RNA Next Generation Sequencing (RNA-seq) combined with exon microarray technology. Interestingly, 16 miRNAs were significantly altered in PD patients compared to healthy controls, whereas 11 miRNAs were modified following DBS treatment compared to controls. Notably, a set of 5 miRNAs (miR-4293, miR-378c, miR-18b*, miR-20a, miR-1249), inverted their trend after DBS treatment, becoming downregulated compared to PD untreated patients’ samples. The group was able to suggest a possible biomarker profile for PD, highlighting specific miRNA changes after DBS [174]. 

Using a different technique (i.e., TLDAs) Cardo and colleagues [175] in 2013 characterized the plasma miRNA profile in 31 PD patients and 25 healthy controls. They validated the best candidates via TaqMan assay and found miR-331-5p upregulated in the plasma of PD patients. MiR-331-5p predicted target genes are implicated in molecular pathways relevant to the CNS, such as axon guidance (i.e., *SRGAP3*, *EPHA4*, *GNAI1* genes). However, the study was based on a limited number of patients at onset stage and requires in the future further replication in larger cohorts of patients at different disease stages [175].

Using the same TLDAs approach, Vallelunga and its group [158] in 2014 analyzed a panel of 754 miRNAs in blood serum samples from PD, MSA patients and healthy donors. They found three miRNAs upregulated in PD patients vs. controls (miR-24, miR-223* and miR-324-3p) and two downregulated (miR-30c and miR-148b). When they analyzed data from MSA patients, they found again the overexpression of miR-24, miR-223* and miR-324-3p, supporting the presence of common features in PD and MSA. Moreover, the expression of the very same miRNAs was significantly different between the two diseases, thus raising the possibility that analysis of a specific subset of circulating miRNAs may be a good way to discriminate between PD and MSA patients, often carrying overlapping clinical features [158]. 

The same year, Botta-Orfila and colleagues [176] analyzed miRNAs in blood serum samples from 10 idiopathic PD (IPD) and 10 familial PD patients carrying the LRRK2 G2019S mutation (LRRK2 PD) vs. 10 controls. By using TaqMan-based miRNA arrays, they observed that miR-19b, miR-29a and miR-29c were significantly reduced in IPD and LRRK2 PD groups. Importantly, this finding was confirmed in a second and in a third validation set. Since the same miRNAs were altered in both PD forms, this reduction was set as a PD common feature. Moreover, the observed downregulation of PD-correlated miRNAs is greater in males than females, fitting with the well-known gender difference found in PD incidence [176,177]. 

In 2015, Fernández-Santiago and collaborators [122] investigated via qPCR the expression levels of the same three miRNAs identified by Botta-Orfila in serum samples collected from idiopathic rapid eye movement (REM) sleep behavior disorder (IRBD) patients, before and after the diagnosis of PD or DLB. They found that miR-19b is downregulated 5 years before the insurgence of PD or DLB, when patients do not show any motor and cognitive symptoms. Therefore, lower miR-19b expression levels may identify IRBD patients who are prone to develop cognitive and motor symptoms. The overlapping association of miR-19b, miR-29a and miR-29c in both PD and DLB could be explained by the fact that both conditions represent the same neurodegenerative disease, even if with different phenotypes. Future studies are needed to validate those results in larger cohorts of patients and miRNAs [122]. 

The same year, Alieva and colleagues [178] analyzed via qPCR the expression of 11 miRNAs in peripheral blood lymphocytes from 20 untreated and 18 treated patients with PD. Two control groups were included in the study: the first one (21 patients) carrying different neurological disorders and the second with 24 healthy controls. miR-9-3p, miR-129, miR-132 miR-9-5p and miR-7 were significantly upregulated in treated PD patients compared to the untreated and the two control groups. Notably, miR-7 was previously found downregulated during PD (see Section 3.1 and Section 3.4). Moreover, consistently with Margis et al., the authors [178] observed that PD treatments led to a change in miRNA expression, which may be monitored to follow both the response to the treatments and the outcome to therapy over time [178].

Following the same experimental strategy, Serafin and colleagues [179] analyzed the expression of miRNAs via qPCR in PB samples from 36 treated and 10 untreated PD patients vs. 10 healthy controls. In this study, miRNAs 30b-5p and 29a-3p were found dysregulated in treated PD patients, albeit opposite to previously published data [171,172]. On the other hand, miR-103a-3p was found for the first time overexpressed in PD treated patients [179]. 

Dong and colleagues [180] performed in 2015 a RNA-seq followed by qPCR validation to analyze serum samples from 169 PD patients vs. 180 controls. The authors were able to identify 4 miRNAs significantly decreased in PD patients [180]. Among this set of miRNAs, miR-146b-5p was reported to be hippocampus-enriched and upregulated in the brains of patients with neuroinflammation [181]. 

Ding and collaborators [182] in 2016 performed again a RNA-seq approach to analyze serum samples from 106 sporadic PD patients and 91 healthy controls. They identified 5 novel miRNAs, one upregulated (miR-195) and four downregulated (miR-185, miR-15b, miR-221 and miR-181a) in PD patients compared to the controls [182].

In 2017, as mentioned in Section 3.2, Chen and collaborators [97] performed a miRNA microarray screening on plasma from 169 sporadic PD patients, 170 healthy controls and 60 essential tremor (ET) patients. The authors identified 31 upregulated miRNAs and 19 downregulated miRNAs. Among those, miR-4639-5p levels were found significantly upregulated in PD patients. Importantly, the elevated miR-4639-5p plasma level was uncorrelated with gender, age of disease onset, L-DOPA treatment and severity of PD motor symptoms, making this miRNA as a potential stable biomarker for early PD diagnosis [97].

Also in 2017, Cao and colleagues [183] performed a validation study on the RNA extracted from exosomes isolated from patients’ serum, via qPCR. Exosomes are nano-sized membrane particles (70–120 nm) secreted by virtually all cells, deriving from the budding of the multivesicular bodies [184]. Recently, exosomes have been identified to actively promote cell-to-cell communication in a health and disease and they are now recognized as natural carriers for RNAs and other molecules [185]. The group of Cao [183] analyzed 24 candidate miRNAs—found dysregulated in serum and CSF of PD patients from previous studies—on 109 PD exosome-derived RNA samples compared to healthy donors. They found that miR-19b was downregulated and miR-195 and miR-24 upregulated in patients with PD compared to controls, raising the possibility that exosomal miRNA profiling in serum may be a novel strategy for the diagnosis of PD [183].

### 5.2. miRNAs in the CSF

CSF sampling, even if invasive, is a valuable source to identify miRNAs potentially useful as PD biomarkers. The first example came from a study performed by Burgos and colleagues in 2014, aimed at profiling the miRNA content in both serum and CSF, in post-mortem samples from 67 PD patients and 78 controls using RNA-seq. Although with the limitation of being performed in post-mortem specimens, this is the first paper to use RNA-seq to compare the miRNA profile in both serum and CSF deriving from the same patients [186]. In PD CSF, 17 miRNAs have been identified as significantly dysregulated, with 6 of those previously identified to be differentially expressed in PD patients: let-7, miR-128, miR-433, miR-485-5p, miR-132, miR-212 [186]. Consistently, miR-132 and miR-212 were found also dysregulated in PD post-mortem prefrontal cortex [187]. On the other hand, 5 miRNAs were differentially expressed in serum PD samples. In particular, miR-16-2-3p, miR-30e and miR-30a-3p were previously found differentially expressed in PD blood specimens (see Section 5.1). It is important to underline that only a minimal overlap was found between the miRNAs identified in CSF with the ones detected in serum. The authors suggested that a further investigation in a living cohort is needed to provide better insights on the interpretation of data as truly valid diagnostic and prognostic indicators for PD [186].

Gui and colleagues [188], evaluated the presence of miRNAs in exosomes from the CSF of PD patients. They profiled the expression of 746 miRNAs by using TaqMan miRNA arrays finding 16 miRNAs upregulated and 11 down regulated in exosomes from CSF of PD patients compared to healthy controls. In particular, miR-1 and miR-19b-3p were significantly downregulated, while miR-153, miR-409-3p, miR-10a-5p and let-7-c-3p were highly overexpressed in PD CSF exosomes—many of them in line with previous studies. Moreover, these miRNAs were found able to target genes involved in crucial pathways for PD, such as neurotrophin signaling and DAergic synapses. To further validate the study, the authors included AD exosomal CSF samples. Interestingly, they found several miRNAs, mRNA transcripts and lncRNAs present in CSF exosomes from both PD and AD patients. In particular, miR-153, miR-409-3p, miR-10a-5p and let-7c-3p were upregulated in PD CSF exosomes vs. AD and control ones, whereas miR-1 and miR-19b-3p were downregulated. These data suggest the potential of a specific subset of miRNAs to distinguish between different neurodegenerative diseases [188].

One year later, Marques and colleagues [189] used qPCR to evaluate the expression levels of 10 miRNAs in CSF patient samples from 28 PD, 17 MSA and 28 healthy controls. They identified two miRNAs differentially expressed in PD vs. controls (miR-24 and miR-205) and four miRNAs in MSA vs. controls (miR-19a, miR-19b, miR-24 and miR-34c). In contrast with [104,158], the authors found an increase of miR-205 and a decrease of miR-24 in the CSF from PD patients compared to controls. A possible explanation could rely on the different nature of the samples used (i.e., blood vs. CSF). However, as for other studies, larger cohorts of patients should be used to validate these results [189].

In conclusion, within the last decade, detecting miRNAs in biological fluids has become a reality. A wide range of studies contributed to give robustness to the methodology, using high throughput techniques and validating the results on larger datasets. Nevertheless, there is still work needed to translate these data into clinics. Although many studies have attempted to identify miRNAs as biomarkers of PD, the results have not always been consistent with each other, especially when comparing post-mortem brain specimens with blood and CSF samples. On the other hand, some consistency was found between different studies performed on liquid biopsies, when comparing PD patients with their controls (see Supplementary Table S1). That is the case of miR-1 (downregulated in PB and CSF-exosomes); miR-30a (upregulated in PB and post-mortem serum); miR-30b (downregulated in PB and PBMC); and miR-195 (upregulated in serum and serum-exosomes). Importantly, let-7g-3p has been found upregulated in CSF-exosomes and CSF-post-mortem in PD patients vs. controls and also in TNF-α treated SHSY-5Y cells, as a model of neuroinflammation linked to PD (see also Section 3.4). Finally, miR-19b has been found downregulated in four different screenings (in serum twice, in serum-exosomes and in CSF) in line also with other reports on MSA (see Section 3.5).

## 6. Development of miRNA-Based Therapies for PD Treatment

We previously discussed how studies performed on post-mortem brains from PD patients are heterogeneous, displaying differences in the disease stage, pathological mutations and pharmacological treatments. To date, no cellular or animal model fully recapitulates the complexity of sporadic PD. For all these reasons, a miRNA-based therapy has not been validated yet, even though different miRNA-based strategies are now being explored for the cure of PD. Furthermore, it is important to underline that a single miRNA has multiple mRNA targets, thus potentially affecting different pathways. Hence, when miRNA-based therapies are proposed, any potential perturbation(s) to other downstream pathways should be carefully analyzed, before being possibly translated into clinic [190,191].

**Figure 3:**
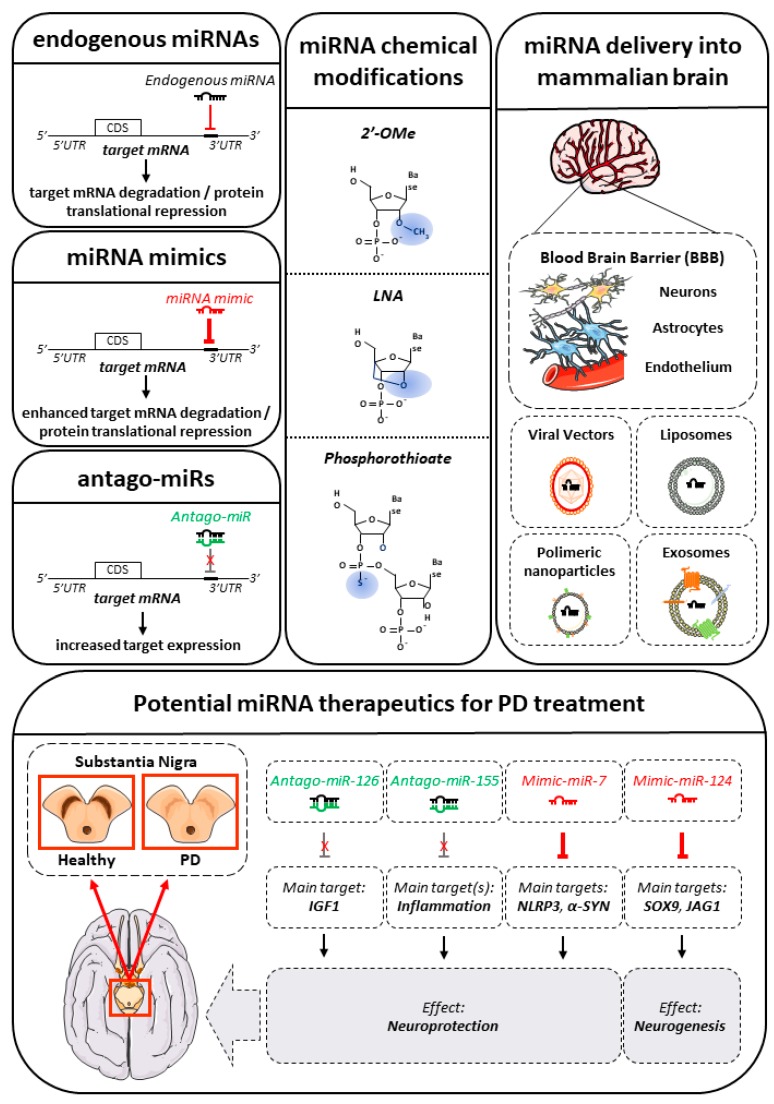
Development of miRNA-based therapies for PD treatment. The three panels on the **top left** show the mechanisms of action of endogenous miRNAs (in black), miRNA mimics (in red) and antago-miRs (in green). Mimics are able to bind their target sequence, thus mimicking the effects of the corresponding endogenous miRNA (in red the inhibitory arrow indicating their effect on the specific target sequence). On the contrary, the antago-miRs bind and block the specific endogenous miRNAs, which are, consequently, unable to bind the target sequence. The **top central** panel shows the most used chemical modifications stabilizing miRNA mimics and antago-miRs backbones. On the **top right** panel the main delivery methods used to carry miRNA mimics and antago-miRs across the BBB (i.e., viral vectors, liposomes, polymeric nanoparticles and exosomes). On the **low-bottom** panel, four miRNAs potentially useful as new therapeutics and their functional effects in the context of PD.

miRNA mimics or anti-miRNAs—called antago-miRs—may represent potential therapeutic tools useful to re-establish miRNAs physiological levels in a pathological condition, such as PD. The different miRNA-based emerging strategies are summarized in Figure 3.

miRNA mimics are synthetic double-stranded RNA molecules that match the mRNA target sequence restoring the miRNA activity in the disease; on the other hand, antago-miRs are single-stranded small RNA molecules, designed to target the miRNAs to be inhibited. Both mimics and antago-miRs can be administered without a delivery vehicle and distributed to various types of tissue [192]. Nevertheless, nucleic acid chemical modifications can improve miRNA stability and delivery efficiency. The most commonly used modifications for miRNAs are: (i) locked nucleic acids (LNA); (ii) 2′-O-methyl modification (2′-O-Me); (iii) phosphorothioate backbones. LNA and 2′-O-Me oligonucleotides contain sugar modifications consisting of the addition of a methylene bridge. The bridge “locks” the sugar in a conformation able to stabilize RNA duplexes. In the LNA, the 2′-OH of ribose is attached to the 5′-carbon atom through the CH_2_ group, while in the case of 2′-O-Me modification, the 2′-hydroxyl group is methylated. The incorporation of LNA-modified nucleotides enhances the stability of the heteroduplex up to 2–10 °C per LNA moiety. On the other hand, the presence of a 2′-O-Me-modified nucleotide into a miRNA increases its binding affinity for the target mRNA in a position-specific manner. In addition, the phosphorothioate backbone decreases any possible nuclease-mediated degradation and increases the membrane permeability [193]. 

The main obstacle when using miRNAs to cure neurodegenerative diseases is represented by the blood-brain-barrier (BBB). One of the strategies to deliver miRNAs into the mammalian brain employs viral vectors, such as the recombinant adeno-associated viruses (rAAV) and lentiviruses. Viral-based gene therapy has attracted increasing interest as a promising therapeutic to treat various diseases, including both genetic and acquired disorders [194]. At the moment, there are a growing number of findings concerning their safety when administered in vivo in both pre-clinical and clinical studies [195,196]. An ongoing phase I/II study on PD patients is based on a lentiviral delivery system to transfer three genes (*ADDC*—aromatic amino acid DOPA-decarboxylase; *TH*—tyrosine hydroxylase; *GTPCH*—GTP-cyclohydrolase-1) called ProSavin. The lentiviral-based drug is injected into the patients’ striatum, in order to reprogram transduced cells to secrete DA. The treatment, although encouraging, showed only little efficacy and the trial is at the moment under examination, waiting for optimal mode and dose of delivery [197,198].

Viral backbones have been recently utilized to deliver miRNAs in vivo. For example, an rAAV vector has been used back in 2010 to carry miR-134 into the postnatal mice brains and resulted in almost 100% transduction efficiency [199]. Interestingly, in 2014 Kim and collaborators, identified miR-126 selectively upregulated in sporadic PD post-mortem neurons (see Section 4). The authors used a lentiviral vector to study the effects of miR-126 overexpression on the IGF-1/PI3K/AKT signaling, a critical pathway for neuronal survival. Using human neuroblastoma SH-SY5Y and rat PC12 cell lines—as DAergic in vitro models—they found that overexpression of miR-126 impaired IGF-1 signaling and increased vulnerability to the PD neurotoxin 6-OHDA. On the contrary, inhibiting miR-126 resulted in increased IGF-1-induced trophic and neuroprotective effects [162]. 

On the other hand, non-viral approaches show high potential, relative safety and easiness for preparation. However, the clinical application of non-viral methods is still restrained by some limitations, including low bioavailability. In order to improve miRNA transfer efficacy several molecular carriers have been developed [200]. Among the non-viral vector systems, the lipid-based carriers represent a valid option for miRNA delivery. They are made of amphiphilic phospholipid bilayers with an aqueous core. A typical liposome is constituted of: (i) cationic lipids (e.g., cholesterol, dioleoylphosphatidyl ethanolamine, or phosphatidylcholine), which electrostatically interact with the polyanions present at the BBB, leading to adsorptive-mediated endocytosis; (ii) neutral lipids, which increase the stability and decrease the toxicity; and (iii) polyethyleneglycol (PEG)-lipids, which form a protective layer over the surface of liposomes, protecting from the binding of plasma proteins. To improve blood-circulation and delivery into the brain, the liposome surface can be modified by the inclusion of different macromolecules, such as polymers, polysaccharides, peptides, antibodies, or aptamers [201]. Following the endocytosis, the liposomes—together with the encapsulated miRNAs—are incorporated into endosomes where specific enzymes destabilize the endosomal membrane allowing the release of their payload [202]. 

In a recent work, Saraiva and collaborators developed a specific polymeric nanoparticles (NPs) formulation, made of poly lactic acid-*co*-glycolic acid (PLGA) and perfluoro-1,5-crown ether (PFCE) and coated with protamine sulphate to form a complex with miR-124 (see Section 3.5). They tested the effect of miR-124 within the brain subventricular zone (SVZ), a principal endogenous niche for adult neurogenesis, found markedly impaired in PD. Looking at the neurogenic and migration potential of SVZ-derived neuroblasts, both in physiological conditions and in a 6-OHDA-mouse model of PD, the authors found that a single administration of miR-124 delivered by NPs, was able to promote a significant increase in the number of neuroblasts reaching the granule cell layer of the olfactory bulb. Moreover, miR-124a targets (i.e., SOX9 and JAGGED1) were remarkable downregulated, as expected [203].

Another powerful way to deliver miRNAs and other small RNAs into the brain is represented by the exosomes. For example, Alvarez-Erviti and collaborators [204] were able to deliver siRNAs to the mouse brain using exosomes. To reduce immunogenicity, they isolated exosomes from the same cell genotype and then they loaded the siRNAs of interest into those exosomes. To deliver the loaded exosomes through the BBB, they engineered dendritic cells to express LAMP2, an exosomal membrane protein, fused with rabies viral glycoprotein (RVG) peptide, able to bind the acetylcholine receptor. The resulting exosomes, injected intravenously, were able to efficiently deliver siRNAs to neurons, microglia and oligodendrocytes. Furthermore, siRNAs were able to inhibit their targets—beta-secretase 1 (BACE1, a well-known AD target within the brain)—at both mRNA and protein levels [204]. 

A more recent work, carried out by Yang and colleagues [205], showed that LAMP2B-modified exosomes loaded with miR-124 mimics were able to cross the BBB reaching the brain, in a focal ischemia mouse model. The ectopic expression of miR-124 in the brain cortex promoted neural differentiation and neurogenesis, attenuating ischemic injury [205]. These data showed that exosomes have the potential to efficiently deliver therapeutic siRNAs and miRNAs into specific organs, including the brain.

Another miRNA that may be relevant to therapy is miR-155, which plays a central role in the microglia inflammatory response to α-SYN in PD, thus being a promising antago-miR-155 for PD (see Section 3.4). Antago-miR-155, loaded either into a peptide with a low pH-induced transmembrane structure or into a NP, was already delivered in two different in vivo lymphoma mouse models, showing a significant reduction in tumor growth, thus suggesting a promising therapeutic for blood tumors [206,207]. Interestingly, a biopharmaceutical company has an ongoing Phase I clinical study for a synthetic LNA-antago-miR-155, named MRG-106. The trial is ongoing on patients with mycosis fungoides-type cutaneous T-cell lymphoma, in order to test antago-miR-155 safety, tolerability and kinetic (ClinicalTrials.gov identifier: NCT02580552).

Furthermore, miR-7, being able to suppress both NLRP3-mediated inflammasome and the nigrostriatal α-SYN (see Section 3.1 and Section 3.4), could be an interesting agonist miRNA with potential neuroprotective effects. Interestingly, a miR-7 mimic has been delivered into mice in the form of a lentiviral construct and proposed as an anti-tumor drug in association with standard chemotherapy [208]. The miRNAs with a potential therapeutic function in modulating PD-related pathways are shown in Figure 3.

## 7. Conclusions and Perspectives

PD is a severe neurodegenerative disease whose incidence increases with aging. The causes and mechanisms of PD are yet not fully clarified but it is assumed that they depend on a complex interaction of genetic susceptibility and environmental factors. Currently, there are no available treatments to block the progression of the disease but only palliative therapies to improve motor symptoms. At this stage, already 70–80% of DAergic neurons are damaged and this makes pharmacological treatments less effective. Hence, the characterization of the molecular mechanisms underlying the onset and progression of PD remains the crucial—and still missing—step to identify new targets and develop novel strategies for the treatment of the disease. In particular, the long preclinical PD phase (estimated to be up to 18 years-long), during which the disease is recognized to progress very slowly, represents a fundamental diagnostic window allowing an early and possibly differential, PD diagnosis. Moreover, this preclinical window can lead to the likely identification of novel potential therapeutic targets involved in early PD modulation. 

Interestingly, the last decade has witnessed the discovery and annotation of thousands of both small and long ncRNAs, which are emerging as key regulators of gene expression in complex organisms. In particular, miRNAs have been found to be involved in the pathogenesis of PD, since almost all PD-related genes resulted regulated by miRNAs. Moreover, a number of miRNAs—whose targets remain to be better characterized—have been suggested to play an active role in PD etiology. 

In addition to their relevance in basic research, miRNAs gained importance as potential biomarkers for the detection of the preclinical stage of the disease. In particular, the use of blood samples for miRNA profiling—easy to extract and to analyze, cheap, allowing patients’ monitoring over time—would represent a step forward to clinical application. A number of promising miRNA candidates have been emerging from the several screenings performed so far and there is growing evidence that miRNA detection can be improved in the next future to become a simple and fast method of diagnosis and prognosis for multifactorial pathologies, such as PD. Notably, the profiling of miRNAs in both serum- and CSF-derived exosomes—where they are stably protected from degradation—bear the potential to become a reliable diagnostic tool for PD in the near future. 

Another important aspect to take into account is the possibility of using miRNA-based approaches for the treatment of PD. Several strategies exist to regulate miRNA levels in vivo. Both miRNA-mimics and antago-miRs are used to modulate miRNAs inside cells. To target PD, miRNAs need to be delivered into the brain, using suitable carriers able to cross the BBB. Once again, exosomes may constitute a natural and innovative solution as miRNA transporters. In fact, when appropriately manipulated, exosomes are able to cross the BBB and enter neurons and other brain cells, where they can release their cargos.

In conclusion, these findings provide novel conceptual and technical advances to better understand PD pathogenesis and, eventually, to identify potential therapeutic strategies to treat this disease. Particularly, the emerging field of miRNAs in neurodegenerative disorders is predicted to deeply affect diagnosis, clinical research and future therapeutic avenues. Understanding the complexity of miRNA regulation in the brain represents a critical goal and a very popular topic in biomedicine, with profound implications for the elucidation of the pathophysiology of major neurodegenerative diseases, including PD. In the long term, shedding light on miRNA-regulated molecular mechanisms can be likely translated into innovative high-clinical-impact therapeutics for PD and other neurodegenerative disorders.

## Supplementary Materials

The following are available online at www.mdpi.com/1422-0067/18/12/2698/s1.
